# The Fitbit One Physical Activity Tracker in Men With Prostate Cancer: Validation Study

**DOI:** 10.2196/cancer.6935

**Published:** 2017-04-18

**Authors:** Erin L Van Blarigan, Stacey A Kenfield, Lucy Tantum, Lisa A Cadmus-Bertram, Peter R Carroll, June M Chan

**Affiliations:** ^1^ Department of Epidemiology and Biostatistics Department of Urology University of California, San Francisco San Francisco, CA United States; ^2^ Department of Urology University of California, San Francisco San Francisco, CA United States; ^3^ Department of Kinesiology University of Wisconsin-Madison Madison, WI United States

**Keywords:** prostatic neoplasms, exercise

## Abstract

**Background:**

Physical activity after cancer diagnosis improves quality of life and may lengthen survival. However, objective data in cancer survivors are limited and no physical activity tracker has been validated for use in this population.

**Objective:**

The aim of this study was to validate the Fitbit One’s measures of physical activity over 7 days in free-living men with localized prostate cancer.

**Methods:**

We validated the Fitbit One against the gold-standard ActiGraph GT3X+ accelerometer in 22 prostate cancer survivors under free-living conditions for 7 days. We also compared these devices with the HJ-322U Tri-axis USB Omron pedometer and a physical activity diary. We used descriptive statistics (eg, mean, standard deviation, median, interquartile range) and boxplots to examine the distribution of average daily light, moderate, and vigorous physical activity and steps measured by each device and the diary. We used Pearson and Spearman rank correlation coefficients to compare measures of physical activity and steps between the devices and the diary.

**Results:**

On average, the men wore the devices for 5.8 days. The mean (SD) moderate-to-vigorous physical activity (MVPA; minutes/day) measured was 100 (48) via Fitbit, 51 (29) via ActiGraph, and 110 (78) via diary. The mean (SD) steps/day was 8724 (3535) via Fitbit, 8024 (3231) via ActiGraph, and 6399 (3476) via pedometer. Activity measures were well correlated between the Fitbit and ActiGraph: 0.85 for MPVA and 0.94 for steps (all *P*<.001). The Fitbit’s step measurements were well correlated with the pedometer (0.67, *P*=.001), and the Fitbit’s measure of MVPA was well correlated with self-reported activity in the diary (0.84; *P*<.001).

**Conclusions:**

Among prostate cancer survivors, the Fitbit One’s activity and step measurements were well correlated with the ActiGraph GT3X+ and Omron pedometer. However, the Fitbit One measured two times more MVPA on average compared with the ActiGraph.

## Introduction

Prostate cancer is the most commonly diagnosed invasive cancer and the second leading cause of cancer death among men in the United States. Emerging evidence suggests that postdiagnosis physical activity may improve clinical outcomes in prostate cancer survivors [1-3]. Our group was the first to observe that men who reported brisk walking and vigorous activity after diagnosis of localized prostate cancer had lower risk of cancer progression and mortality [2,3]. Like most cohort analyses of cancer survivors, however, these studies relied on self-reported physical activity. Self-report assessments are subject to limitations including poor ability to measure low-intensity or unstructured activities and lack of precision for quantifying activity intensity or duration [4]. Therefore, objective measures of physical activity are needed to better inform guidelines for prostate cancer survivors. No physical activity tracker has been validated for use in cancer survivors.

Whereas research-grade accelerometers (eg, the ActiGraph) are used in some cancer survivorship studies, they are costlier than consumer-based physical activity trackers and are generally not acceptable to wear over periods longer than 1 week. Leveraging commercial wearable devices may enable more research teams to capitalize on the advantages of objective measurement. These devices are appropriate for long-term measurement of behavior and may be useful tools as part of a physical activity intervention [5-7].

A growing number of consumer-level, wearable physical activity trackers may be well-suited for both objectively measuring physical activity and promoting vigorous-intensity activity in men with prostate cancer. These devices have many advantages for health research, including low participant burden, lack of reliance on accurate recall, and the ability to upload individual-level physical activity data to a cloud-based database, allowing both users and researchers to view data in real time. Previous studies have reported that modern physical activity trackers provide a valid measure of physical activity in controlled laboratory settings, but few studies have evaluated such trackers in free-living conditions [8]. This is important because the type and intensity of physical activity (eg, gait speed) for chronic disease populations may differ from the types of activity typically assessed in a lab-based validation study. Moreover, there is a lack of data on the validity of physical activity trackers in older populations and cancer survivors, who engage in different types of activities compared with younger adults.

Although several manufacturers make consumer-based physical activity trackers, Fitbit is the dominant brand used by health behavior researchers. Fitbit makes several models of physical activity trackers. We selected the Fitbit One for this study because it was one of the most advanced Fitbit models available in 2013; it remains a widely available and popular tracker in 2017. The Fitbit One is a 3-axis, accelerometer-based, physical activity tracker that measures steps, floors climbed, distance traveled, calories burned, physical activity, and sleep. The device is small (0.76”×0.38”×1.89”) and can be clipped to a belt, tight-fitting clothing, or a pocket.

In this study, we validated the Fitbit One’s measures of physical activity over 7 days in free-living men with localized prostate cancer against the ActiGraph GT3X+ accelerometer (gold standard) and a physical activity diary. We also compared the devices’ measures of steps with the HJ-322U Tri-axis USB Omron pedometer. We hypothesized that the Fitbit One would provide a valid measure of physical activity in men with prostate cancer.

## Methods

### Study Population

This study was conducted among 25 men with prostate cancer. Participants were recruited in the University of California, San Francisco (UCSF) Department of Urology between 2013 and 2015. To be eligible, the men must have been diagnosed with adenocarcinoma of the prostate and be on active surveillance. We excluded 2 men with missing ActiGraph GT3X+ accelerometer data (our gold standard) and 1 man missing Fitbit data, leaving 22 men available for analysis. All participants provided written informed consent, and this study was approved by the UCSF Institutional Review Board.

### Physical Activity Assessments

Participants were asked to wear 3 physical activity trackers—ActiGraph GT3X+ (ActiGraph Inc, United States), Fitbit One (Fitbit Inc, United States), and HJ-322 Tri-axis Omron pedometer (Omron Healthcare, The Netherlands)—on a belt around their waist and keep a physical activity diary for 7 consecutive days.

The ActiGraph GT3X+ is considered the gold standard for activity tracking; it has been validated in numerous populations and is widely used in research settings [9,10]. The Omron pedometer is also widely used in research and has also been validated in healthy populations [11]. The trackers were positioned next to one another on a belt over the right hip. Participants were instructed to wear the belt during all waking hours and to remove the belt when sleeping, bathing, or swimming. All setup, charging, and syncing of the devices was done by the research staff. Participants were not provided with the Fitbit One charging cable or wireless sync dongle and were instructed not to change the devices’ setup. Therefore, the only feedback that the participants may have received while wearing the devices was their daily steps on the pedometer and the Fitbit One. Fitbit Inc donated the Fitbit One devices used in this study but had no role in the design, conduct, or analysis of the study.

The physical activity diary has been previously described [4]. Participants were provided with seven 24-hour charts that included one row for each hour of the day and were asked to report how many minutes they spent in each of the following activities during each hour: lying down or sleeping; walking outdoors (eg, for exercise, transport); mixed standing and walking at home; mixed standing and walking away from home (eg, work, shopping); sitting at home; sitting at work or in a car or train; sports or other activities. For sports or other activities, the participants were asked to specify the type of activity (eg, tennis, swimming, yoga, gardening) and the intensity of the activity (eg, low, medium, high). If they participated in weight lifting, they were asked to indicate the muscle group worked (eg, arms, legs, back).

### Data Processing

The accelerometer data were processed using ActiLife version 6.13.3 (ActiGraph, LLC). The data were downloaded in 5-second epochs. Nonwear was defined as an interval of consecutive 0 counts lasting 60 minutes or longer. A valid day was defined as a minimum of 10 hours of wear; we required at least three valid days. A total of 2 men were missing their ActiGraph GT3X+ accelerometer data: 1 man did not wear the belt on the instructed days, and the data for the other man did not download correctly. For the remaining men, we used the ActiGraph GT3X+ data to identify valid calendar days for all devices and the diary. We used the Troiano cut-points to estimate duration of light, moderate, and vigorous physical activity from the accelerometer data: light activity was defined as 100-2019 counts per minute, moderate activity was defined as 2020-5998 counts per minute, and vigorous activity was defined as 5999 or more counts per minute [12].

The Fitbit One devices were synced by the research staff to the manufacturer’s website and the available data were downloaded using the “export your data” function under settings for analysis [13]. The data available for each participant included daily total steps, minutes lightly active, minutes fairly active, and minutes very active, as well as other variables not examined (eg, estimated calories burned, distance, floors). On the basis of the information from the Fitbit website [14] and data reported in a recent validation study of the Fitbit Flex [15], Fitbit trackers calculate active minutes using metabolic task equivalents (METs). For example, “fairly active” minutes correspond to minutes engaged in activities requiring 3-5.9 METs. Therefore, we assumed that “light,” “fairly active,” and “very active” physical activity categories in the Fitbit data corresponded to standard definitions of light (<3 METs), moderate (3-5.9 METs), and vigorous (≥6 METs) physical activity, respectively [16].

The Omron pedometer devices were synced to the manufacturer’s website by the research staff and the available step data were downloaded for analysis [17].

For the diary data, we used the compendium of physical activities to assign specific MET values to each of the activities reported by the participants. Activities were then categorized as vigorous (≥6 METs), moderate (3-5.9 METs), or light (<3 METs; see Table 1) [16]. We summed the duration of time in each of the activity categories to obtain estimates of time spent in light, moderate, and vigorous physical activity.

**Table 1:** Physical activities reported in a 7-day physical activity diary by 20 men with localized prostate cancer.

Activity		n^a^ (%)	Mean (SD), minutes per day^b^	MET^c^ value [16]
**Moderate-intensity activities (MET 3-5.9)**					
	Walking	19 (95)	29 (23)	3.5
	Golf	6 (30)	87 (55)	3.5
	Heavy outdoor work	3 (15)	65 (92)	5.5
	Other aerobic activities	5 (25)	15 (12)	4.3
	Gardening	6 (30)	11 (12)	3.5
	Weight lifting	5 (25)	10 (6)	3.5
	Housework	4 (20)	13 (11)	3.3
	Rowing	1 (5)	13 (-)^d^	4.8
	Elliptical	2 (10)	9 (2)	5.0	
	Hiking	1 (5)	32 (-)^d^	5.3
	Yoga	3 (15)	8 (5)	3.3
**Vigorous-intensity activities (MET ≥6)**					
	Bicycling	7 (35)	36 (39)	6.8
	Tennis	3 (15)	90 (130)	7.3
	Jogging	3 (15)	9 (7)	7.0
	Running	3 (15)	11 (7)	9.8

^a^Two of the 22 men in this study did not complete a physical activity diary.

^b^Average minutes per day spent engaged in that activity among men who ever reported that particular activity.

^c^MET: metabolic task equivalent.

^d^Only one man reported rowing or hiking, so we did not calculate a standard deviation.

### Statistical Analysis

In order to calculate average daily minutes of light, moderate, and vigorous physical activity for each device, we summed the total number of minutes per day across days with valid data and divided it by the number of valid days. We then used descriptive statistics (eg, mean, standard deviation, median, interquartile range [IQR]) and boxplots to examine the distribution of average daily light, moderate, and vigorous physical activity and steps measured by each device and the diary. Average daily light and moderate physical activity and steps were normally distributed; average daily vigorous activity was skewed right. Therefore, we used Pearson correlation coefficients to compare measures of light and moderate physical activity and steps, and Spearman rank correlation coefficients to compare measures of vigorous activity between devices and the diary. All statistical analyses were performed using SAS v9.4 (SAS Institute, Inc) and two-sided *P* values <.05 were considered statistically significant.

## Results

On average, the men had 5.8 days of valid wear time available for analysis. The activities reported by men with prostate cancer in our study are presented in Table 1. Consistent with the prior publications reporting activity in men with localized prostate cancer [2,3], walking was the most common form of exercise, reported by 19 out of the 20 men with diary data (95%). Cycling was the next most popular activity, reported by 7 out of 20 men (35%).

Sociodemographic and clinical characteristics of the study participants are presented in Table 2. The median age was 66 years (IQR 56-83 years) and median body mass index (BMI) was 26.7 kg/m^2^ (IQR 20.1-34.4 kg/m^2^). Of the 22 men, 15 were white (68%), 6 (27%) reported “other race,” and 1 man (5%) was Asian or Pacific Islander. The median time from diagnosis to enrollment was 1.6 years (IQR 0.7-3.5).

**Table 2:** Characteristics of the 22 men with localized prostate cancer, who wore a Fitbit One, ActiGraph GT3X+, and Omron Pedometer, and kept a physical activity diary for 7 days.

Characteristics		Median (IQR) or n (%)
Age (years), median (IQR)		66 (56-83)
Body mass index (kg/m^2^), median (IQR)		26.7 (20.1-34.4)
**Race, n (%)**		
	White	15 (68)
	African American	0 (0)
	Asian or Pacific Islander	1 (5)
	Other	6 (27)
Years since diagnosis, median (IQR)		1.6 (0.7-3.5)
Prostate-specific antigen at diagnosis (ng/ml), median (IQR)		5.6 (0.7-17.0)
**Gleason score, n (%)**		
	6	19 (86)
	3+4	3 (14)
**Clinical T-stage, n (%)**		
	T1c	17 (77)
	T2a	5 (23)

The physical activity trackers and diary detected different absolute levels of light, moderate, and vigorous physical activity (Table 3). The mean (SD) daily vigorous physical activity measured by each device and diary was: 19 (20) minutes/day according to the Fitbit One, 4 (6) minutes/day according to the ActiGraph GT3X+, and 29 (59) minutes/day according to the diary. For moderate activity, the values were: 81 (37) minutes/day according to the Fitbit One, 47 (26) minutes/day according to the ActiGraph GT3X+, and 80 (62) minutes/day according to the diary. Combined, the Fitbit One measured an average of 49 more minutes of MVPA per day than the ActiGraph GT3X+. However, this difference varied substantially within individuals, ranging from −5 minutes (ie, the Fitbit measured 5 minutes less MVPA than the ActiGraph GT3X+) up to 109 minutes. Finally, the Fitbit One recorded 190 (50) minutes/day and the ActiGraph GT3X+ recorded 125 (32) minutes/day on average of light activity. The average daily step counts were: 8724 (3535) according to the Fitbit One, 8024 (3231) according to the ActiGraph GT3X+, and 6399 (3476) according to the Omron pedometer.

**Table 3:** Average duration of daily physical activity and steps measured by the Fitbit One, ActiGraph GT3X+, Omron pedometer, and a physical activity diary over 7 days among 22 men with localized prostate cancer.

Measuring device	Fitbit One		ActiGraph GT3X+		Diary^a^		Omron pedometer^a^	
No. of men	22		22		20^b^		21^b^	
Activity category	Mean (SD)	Median (range)	Mean (SD)	Median (range)	Mean (SD)	Median (range)	Mean (SD)	Median (range)
Vigorous^c^ (minutes/day)	19 (20)	11 (1-63)	4 (6)	1 (0-27)	29 (59)	9 (0-240)	-	-
Moderate^d^ (minutes /day)	81 (37)	76 (13-173)	47 (26)	39 (14-113)	80 (62)	64 (9-195)	-	-
MVPA^e^ (minutes/ day)	100 (48)	92 (17-184)	51 (29)	42 (15-116)	110 (78)	77 (16-266)	-	-
Light^ef^ (minutes /day)	190 (50)	185 (92-283)	125 (32)	121 (72-185)	-	-	-	-
Steps	8724 (3535)	8032 (2729-15,843)	8024 (3231)	7031 (4207-15,251)	-	-	6399 (3476)	5051 (1362-12,532)

^a^The diary and pedometer did not measure all activity categories of interest. The diary did not measure light activity or steps. The pedometer does not measure light, moderate, or vigorous physical activity.

^b^Two men did not complete a physical activity diary and there were no data on one of the pedometers after it was returned by the participant.

^c^Vigorous activity included 6+ metabolic task equivalent (MET) activities (cycling, jogging, running, tennis).

^d^Moderate activity included 3-5.9 MET activities (heavy outdoor work, elliptical, gardening, hiking, housework, weight lifting, other aerobic activities, rowing at a moderate pace, walking, and yoga).

^e^ MVPA: moderate-to-vigorous physical activity.

^f^Light activity included activities with <3 MET values (eg, easy walking).

Despite differences in the absolute levels of activity and steps recorded, average daily vigorous, moderate, and light activity and steps were highly correlated between the trackers (Table 4). Comparing the Fitbit One and ActiGraph GT3X+, the correlation coefficients were: .65 for vigorous activity, .70 for moderate activity, .72 for light activity, and .94 for steps (all *P*<.001). The Fitbit One and the ActiGraph both recorded step measurements that were relatively well correlated with the pedometer (.67 and .72, respectively; *P*<.001).

**Table 4:** Correlation coefficients comparing average daily vigorous, moderate, moderate-to-vigorous, and light physical activity and steps measured by the Fitbit One, ActiGraph GT3X+, Omron pedometer, and a physical activity diary among 23 men with localized prostate cancer.

Measuring device^a,b^	Fitbit One versus ActiGraph GT3X+		Fitbit One versus Diary^c^		Fitbit One versus Omron pedometer		ActiGraph GT3X+ versus Diary		ActiGraph GT3X+ versus Omron pedometer	
No. of men	22		20		21		20		21	
Activity Category	*r*	*P* value	*r*	*P* value	*r*	*P* value	*r*	*P* value	*r*	*P* value
MVPA	.85	<.001	.84	<.001	-	-	.68	.001	-	-
Vigorous activity	.65	.001	.47	.04	-	-	.66	.001	-	-
Moderate activity	.70	<.001	.38	.10	-	-	.57	.009	-	-
Light activity	.72	<.001	-	-	-	-	-	-	-	-
Steps	.94	<.001	-	-	.67	.001	-	-	.72	<.001

^a^Pearson correlations were used for measures that were normally distributed: steps, light activity, and moderate activity. Spearman correlations were used for skewed measures: vigorous activity.

^b^Correlation coefficients were not calculated if one of the devices did not measure the activity category of interest. The diary did not measure light activity or steps. The pedometer does not measure light, moderate, or vigorous physical activity.

^b^One individual reported high levels of tennis, which was classified as a vigorous activity in the diary data based on the standard MET value for tennis (7.3 METs) but was classified as a moderate activity for this individual by the Fitbit. The correlations for vigorous and moderate physical activity as assessed by the Fitbit One and diary excluding this individual were *r*=.61, *P*=.006 and *r*=.61, *P*=.005, respectively. We also reclassified tennis as a moderate activity in the diary data, and the correlations between the Fitbit One and the diary for vigorous and moderate activity were similarly improved (*r*=.58, *P*=.008 and *r*=.73, *P*<.001).

In contrast to the ActiGraph GT3X+, the Fitbit One’s measures of average moderate and vigorous physical activity were not well correlated with the physical activity diary (*r*=.47, *P*=.04 and *r*=.38, *P*=.10). Upon examining scatterplots, we identified one participant who had a low measure of vigorous activity (5 minutes/day) and a high measure of moderate activity (173 minutes/day) according to the Fitbit One, but high measure of vigorous activity (240 minutes/day) and a low measure of moderate activity (26 minutes/per day) according to the diary. This discrepancy appeared to be due to a difference in the classification of the intensity of tennis between the Fitbit and diary data. The individual reported an average of 240 minutes per day of tennis and 26 minutes per day of walking. Based on the compendium of MET values [16], tennis requires 7.3 METs and was thus classified as a vigorous activity in the diary data. We ran two sensitivity analyses to evaluate the impact of this individual on the estimated correlation coefficients. First, we calculated correlations between vigorous and moderate physical activity as assessed by the Fitbit One and diary excluding this individual (*r*=.61, *P*=.006 and *r*=.61, *P*=.005, respectively), and classifying tennis as a moderate activity in the diary data (*r*=.58, *P*=.008 and *r*=.73, *P*<.001).

## Discussion

### Principal Findings

In this study, we validated the Fitbit One’s measure of physical activity and steps against the ActiGraph GT3X+, Omron pedometer, and a 7-day physical activity diary in free-living conditions among 22 men with localized prostate cancer. The Fitbit One’s measure of vigorous, moderate, and light physical activity and steps were well correlated with the ActiGraph GT3X+ accelerometer, Omron pedometer, and physical activity diary. Furthermore, the mean time spent engaged in moderate and vigorous activity was comparable between the Fitbit One and physical activity diary, but substantially more than the estimated amount of time in these activities recorded by the ActiGraph GT3X+.

Six studies have demonstrated the validity of the Fitbit One in laboratory settings, but only one prior study has validated the Fitbit One’s measure of physical activity in free-living conditions [8,18]. In that study, 21 healthy Australian adults (mean age: 33 years) wore 7 consumer-level activity monitors, including the Fitbit One, and two research-grade monitors, the ActiGraph GT3X+, and BodyMedia SenseWear Model MF (BodyMedia Inc, United States) for 48 hours. Consistent with our study protocol, the Fitbit One and ActiGraph GT3X+ were both worn on the right side of the waist on an elastic belt. In that study, the correlation between the Fitbit One and ActiGraph GT3X+ measures of MVPA was .91. The ActiGraph GT3X+ measured a median of 58.5 minutes of MVPA and the median absolute difference between the Fitbit One and ActiGraph GT3X+ measures of MVPA was 58.6 minutes. Our findings among older men with prostate cancer were remarkably consistent with those reported by Ferguson et al [18]. The median absolute difference between the Fitbit One and the ActiGraph GT3X+ measures of MVPA was 47 minutes in our study population. Overall, it appears that the Fitbit One’s measure of MVPA is well correlated with the ActiGraph GT3X+, a widely accepted gold standard research-grade accelerometer. However, the Fitbit One may overestimate MPVA in free-living young and older adult populations.

A novel aspect of our study was the inclusion of a physical activity diary. Interestingly, the Fitbit One’s measures of average daily moderate and vigorous physical activity were very similar to the time engaged in these activities reported in the participants’ diaries. On average, the Fitbit One and diary measured 100 and 110 minutes/day of MVPA, respectively. In contrast, the ActiGraph GT3X+ measured an average of 51 minutes of MVPA per day. Proportionally, there was a larger discrepancy in vigorous than moderate activity. The ActiGraph GT3X+ measured an average of 4 minutes per day, the Fitbit One measured an average of 19 minutes per day, and the diary reported an average of 29 minutes per day of vigorous activity.

### Limitations

On the basis of the diary data, we observed that walking and cycling were the most popular physical activities among men with localized prostate cancer, followed by golf and outdoor work or gardening. The distribution of time spent in various activities among the men in our study was consistent with data reported from two large cohort studies of men with prostate cancer in the United States [2,3]. Of note, none of the participants in our study swam for exercise; Fitbit, Inc instructs users not to swim with Fitbit One trackers. Newer models of activity trackers have waterproof features as well as include algorithms to determine what type of activity is being done, and future research is needed to assess the accuracy and utility of such data.

In addition, a limitation of self-reported physical activity data is the lack of objective assessment of activity intensity. Our data clearly demonstrated this point when comparing the Fitbit One and diary measures of moderate and vigorous physical activity. One participant reported a high duration of tennis, which we classified as vigorous activity based on the compendium of MET values. However, based on the Fitbit One’s data, it appeared that tennis was better classified as a moderate intensity activity for this individual. It is also possible that, when worn on the hip, the Fitbit One and ActiGraph GT3X+ are not very good at detecting the intensity of a sport that requires a lot of arm motion. Future studies should combine objective and self-reported physical activity data in order to best assess the participants’ usual duration, intensity, and mode of physical activity.

This study had a number of strengths, including: (1) comparing the Fitbit One to the ActiGraph GT3X+ and a physical activity diary, which are widely accepted gold standard measures; (2) having participants wear the monitors and keep a diary in free-living conditions for 7 consecutive days; and (3) being the first study to examine the validity of the Fitbit One in cancer survivors. Lack of generalizability to populations with different racial or ethnic, sex, and disease status, or physical activity patterns is a potential limitation of our study, although our results were markedly similar to the prior validation study conducted in healthy young adult men and women.

### Future Work

New physical activity trackers, as well as updated products and software, are constantly being released. At the time this study was initiated in early 2013, the Fitbit One was the most advanced Fitbit model available. Since that time, several new models have been released, including wrist-based trackers. Further studies are needed to determine whether wrist-based trackers have accuracy similar to the Fitbit One, as well as evaluate patient preferences between a clip-on versus wrist-based device. Overall, rigorously evaluating and reporting results in peer-reviewed journals on up-to-date and relevant devices is a particular challenge for researchers in this field due to the fast growth of the activity tracker industry. Nonetheless, validation studies are crucial for the design and interpretation of clinical studies utilizing wearable physical activity trackers.

### Conclusions

In conclusion, the Fitbit One’s measure of physical activity and steps are well correlated with the ActiGraph GT3X+, Omron pedometer, and a physical activity diary. The Fitbit One’s estimate of time spent in MVPA was consistent with that reported in the physical activity diary, but approximately twice the duration, on average, measured by the ActiGraph GT3X+. Therefore, the absolute duration of moderate and vigorous activity measured by the Fitbit One and self-reported methods should be interpreted cautiously.

## Abbreviations

BMI: body mass index
IQR: interquartile range
MET: metabolic task equivalents
MVPA: moderate-to-vigorous physical activity
PSA: prostate-specific antigen
SD: standard deviation
UCSF: University of California, San Francisco
